# Novel mutations and the ophthalmologic characters in Chinese patients with Wolfram Syndrome

**DOI:** 10.1186/s13023-019-1161-y

**Published:** 2019-08-07

**Authors:** Youjia Zhang, Lili Feng, Xiangmei Kong, Jihong Wu, Yuhong Chen, Guohong Tian

**Affiliations:** 1 Department of Ophthalmology and Visual Science, Eye, Ear, Nose and Throat Hospital, Shanghai Medical College, Fudan University, Shanghai, China, 83 Fenyang Road, Shanghai, 200031 China; 2 State Key Laboratory of Medical Neurobiology, Institutes of Brain Science and Collaborative Innovation Center for Brain Science, Eye Ear Nose and Throat Hospital of Fudan University, 83 Fenyang Road, Shanghai, 200031 China; 3 NHC Key Laboratory of Myopia (Fudan University), Laboratory of Myopia, Chinese Academy of Medical Sciences, Eye Ear Nose and Throat Hospital of Fudan University, 83 Fenyang Road, Shanghai, 200031 China; 4 Shanghai Key Laboratory of Visual Impairment and Restoration (Fudan University), Eye Ear Nose and Throat Hospital of Fudan University, Shanghai, China, 83 Fenyang Road, Shanghai, 200031 China

**Keywords:** Wolfram syndrome, DIDMOAD, Optic atrophy, Next generation sequence, *WFS1*, *CISD2*

## Abstract

**Background:**

Wolfram Syndrome (WFS) is a rare autosomal recessive neurodegenerative disease which has a wide spectrum of manifestations including diabetes insipidus, diabetes mellitus, optic atrophy and deafness. *WFS1* and *CISD2* are two main causing genes of WFS. The aim of this study was to illustrate the ophthalmologic manifestations and determine the genotype of Chinese WFS patients.

**Results:**

Completed ophthalmic examinations and family investigations were performed on 4 clinically diagnosed WFS patients from 4 unrelated families. Genetic testing was done by the next generation sequencing of candidate genes. One patient carried a homozygous mutation (c.272_273del) in *CISD2*, two patients carried compound heterozygous mutations (c.1618 T > G + c.2020G > A and c.1048 T > A + c.2020G > A) in *WFS1*, and one patient carried a heterozygous mutation (c.937C > T) in *WFS1*. Three of them were novel mutations.

**Conclusions:**

Our study indicated WFS in Chinese is a neurodegenerative disease with both wide spectrum of clinical features and genetic heterogeneity. We found three novel mutations in WFS patients, and to our best knowledge, this is the first report of Chinese WFS patient with mutation in *CISD2*.

**Electronic supplementary material:**

The online version of this article (10.1186/s13023-019-1161-y) contains supplementary material, which is available to authorized users.

## Background

Wolfram syndrome (WFS; MIM #222300), first described in 1938 by Wolfram and Wagener, is a rare hereditary autosomal recessive disease. The prevalence of WFS was estimated to be 1 in 770,000 in UK [1] and 1 in 710,000 in the Japanese population [2]. As a progressive neurodegenerative disorder, WFS has a wide spectrum of clinical manifestations. The main phenotypes of WFS are diabetes insipidus (DI), diabetes mellitus (DM), optic atrophy (OA) and deafness (D) [1, 3]. Around 50% patients harbor all these manifestations, so WFS was also referred to as the acronym DIDMOAD syndrome [1, 3, 4]. Other common manifestations include neurologic and psychiatric disorders, renal tract abnormalities, endocrine disorders, as well as many others [5]. The major diagnosis criterion of WFS is the coincidence of early onset type 1 DM and bilateral OA before the second decade [1, 5–7]. Because of the multi-system neurodegeneration, the prognosis of WFS is very poor and the patients’ median life expectancy is about 30 years (range 25–49 years) [1].

*WFS1* on chromosome 4 is the causative gene of Wolfram Syndrome type 1 (WFS1) [8], and the loss-of-function mutations of *WFS1* have been identified in most of patients with WFS [5, 9]. *WFS1* encodes wolframin, an endoplasmic reticulum (ER) transmembrane protein [10]. Wolframin is widely expressed in neurons, pancreas, heart, muscle, liver, spleen and kidney [11]. It has also been detected in optic nerve glial cells and retinal ganglion cells [12, 13]. The main function of wolframin are reducing the ER stress, maintaining the Ca^2+^ homeostasis and regulating the biosynthesis and secretion of insulin [14–16].

In addition, mutations of *CISD2* are responsible for Wolfram Syndrome type 2 (WFS2; MIM #604928), which has variant features including gastrointestinal ulceration and bleeding tendency without diabetes insipidus [17–19]. *CISD2*, CDGSH iron-sulfur domain-containing protein 2, located on chromosome 4q22–24, encodes endoplasmic reticulum intermembrane small protein (ERISP) [19]. Although the biological functions of *CISD2* still remain incompletely defined, some studies show that it has a similar role with *WFS1* in maintaining the homeostasis of Ca^2+^ and ER and the cross-talk between ER and mitochondria [20, 21].

In this study, we performed a clinical and genetic investigation on 4 unrelated Chinese patients with WFS. We systematically reviewed their clinical ophthalmologic features and identified 3 novel mutations in *WFS1* and *CISD2* gene. And we reported the first Chinese patient with WFS2 carried a homozygous mutation in *CISD2*.

## Patients and methods

### Patients

We retrospectively reviewed 4 consecutive patients diagnosed with WFS at Ophthalmology Department of Eye Ear Nose and Throat Hospital of Fudan University from 2013 to 2018. This study was approved by the Eye Ear Nose and Throat Hospital of Fudan University Institutional Review Board, and written formal consent was obtained from all enrolled patients or their legal guardians. Patients were enrolled in our study when meeting one of the following two criteria: 1) the early onset DM and progressive OA, not explained by any other diseases; 2) the identification of 2 pathological *WFS1*/*CISD2* mutations. DM was diagnosed by WHO criteria [22]. OA was confirmed by funduscopic examination of the optic nerve head with pallid appearance and by the evidence of atrophy of the peripapillary nerve fiber layer on optical coherence tomography (OCT). Magnetic resonance imaging (MRI) or computed tomography (CT) scan was also utilized to exclude compressive optic neuropathies. 110 healthy Chinese people, without diagnosis of DM, OA or any other serious ocular or systematic diseases, were also included in this study.

### Clinical investigation

All patients underwent a complete ophthalmologic examination, including visual acuity (VA) examination, intraocular pressure measurement, slit-lamp biomicroscopy, ophthalmoscope, visual fields assessment (Carl Zeiss Meditec, Inc., Dublin, CA, United States), electroretinography (ERG) and visual evoked potentials (VEP) (LKC UTAS E3000 LKC Technologies, Inc., United States). The OCT (Cirrus OCT 5000, Carl Zeiss Meditec, Inc., Dublin, CA, United States) was performed for each patient to evaluate retinal nerve fiber layer (RNFL) thickness. The MRI was performed in 2 patients and CT scan was completed in the other 2 patients. The audiological, urological, neurological and psychiatric examinations results were recorded from the medical records.

### Genetic analysis

Genomic DNA samples were extracted from whole blood samples of the patients, their relatives, and 110 healthy Chinese people. Genetic testing was performed in all four patients by next generation sequence (NGS). A panel including 790 ophthalmology associated genes were sequenced by Illumina HiSeq 2000 (Illumina, Inc., San Diego, CA, United States) sequencing system. The average depth was 200x. Family members of the probands were validated by Sanger sequence.

The detected mutations were checked in 110 Chinese normal controls by Sanger Sequence. Conservation of the mutation sites was evaluated by Clustal Omega [23]. Polymorphism Phenotyping 2 (PolyPhen2) [24] and Sorting Intolerant from Tolerant (SIFT) [25] were applied for the assessment of pathogenicity of detected mutations.

## Results

### General clinical manifestations

Four Chinese WFS patients from 4 different families were enrolled in our study. The demographic and clinical features of the 4 patients are shown in Table 1. All patients were male. The median age of patients was 25 years (range 11–42 years). Patient 1 came from consanguineous family and has an elder brother diagnosed of DM at age of 10 years and died from ketosis encephalopathy at age of 17 years. Patient 2 has an elder sister diagnosed of WFS with same symptoms.

**Table 1:** Clinical characters of patients with wolfram syndrome

Case no.	Age	Sex	Family history	DM, age of diagnosis	Presenting age of impaired vision	OA, age of diagnosis	DI, age of diagnosis	HI, age of diagnosis	Other features, age of diagnosis	BCVA
1	11 years	M	Positive * (brother)	Type I, 9 years	Bilateral, 9 years	Bilateral, 10 years	Central DI, 11 years	Bilateral HF, 11 years	Abnormal MRI of brain,*** 11 years Abnormal EEG, 12 years	OD: 20/400 OS: 20/400
2	26 years	M	Positive ** (sister)	Type I, 10 years	Bilateral, 7 years	Bilateral, 7 years	No	No	No	OD: 20/400 OS: 20/400
3	42 years	M	Negative	Type I, 28 years	Bilateral, 39 years	Bilateral, 42 years	No	Bilateral HF	No	OD: 20/60 OS: 20/100
4	24 years	M	Negative	Type I, 10 years	Bilateral, 15 years	Bilateral, 24 years	No	Bilateral sensorineural deafness, 2.3 years	Left-sided glaucoma, 24 years	OD: 20/25 OS: 20/400

*DM* Diabetes Mellitus, *OA* Optic Atrophy, *DI* Diabetes Insipidus, *HI* Hearing Impairment, *HF* High-frequency Hearing Impairment, *BCVA* Best Corrected Visual Acuity, *EEG* Electroencephalography, *OD* right eye, *OS* left eye. *Patient 1 had a brother who acquired diabetes mellitus at around 10 years old and died at 17 years old with ketosis encephalopathy. **Patient 2 has a 27 years old sister who was diagnosed with DM at 11 years old and had poor visual acuity since 16 years old. ***Patient 1 showed the absence of the physiological high signal of the posterior pituitary gland on T1WI of cranial MRI

All patients presented to our ophthalmology clinic because of progressive loss of vision (Table 1). They all had the coincidence of DM and OA. The median age at DM onset was 10 years (range 9–28 years). Three of them had various degree of hearing impairment: two patients had bilateral high-frequency hearing impairment and one had bilateral sensorineural deafness. Patient 1 was diagnosed as central diabetes insipidus by the water deprivation and desmopressin challenge test, and he also showed absence of the physiological high signal of the posterior pituitary gland on T1-weighted images of cranial MRI and an abnormal electroencephalography (EEG). No patient presented renal tract abnormalities, psychiatric diseases or behavioral disorders.

### Ophthalmologic manifestations

**Fig. 1:**
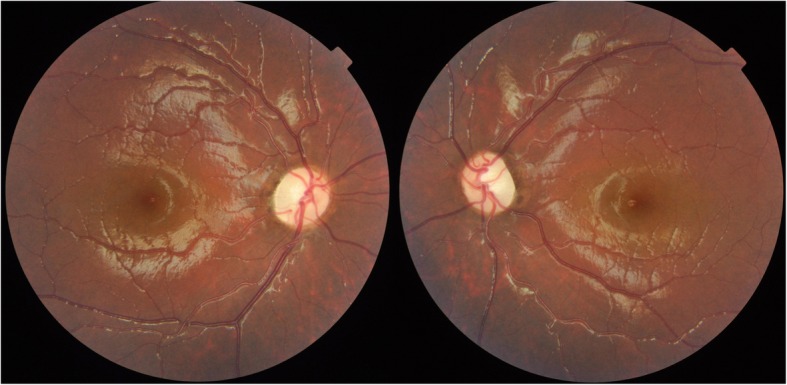
Fundus photography of the patient with *CISD2* mutation. Fundus photography shows optic disc diffused pallid bilateral without diabetes retinopathy

**Fig. 2:**
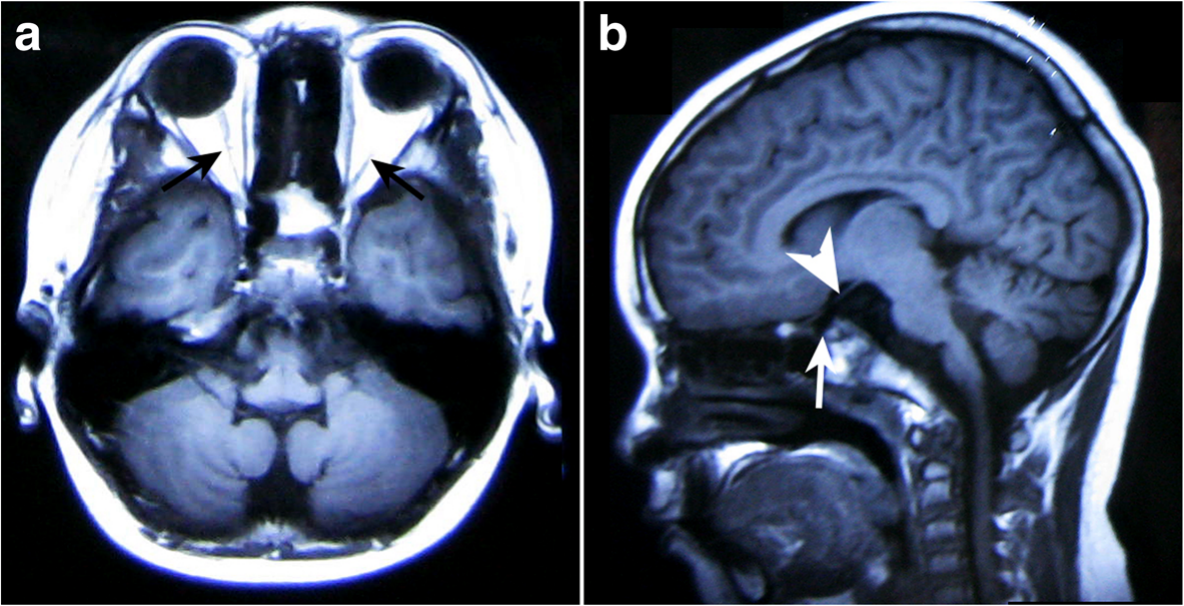
The magnetic resonance imaging (MRI) of the patient with *CISD2* mutation. **a**: axial T1WI shows bilateral atrophy of optic nerves (black arrows) and (**b**): sagittal T1WI shows absence of the physiological high signal of the posterior lobe of the pituitary gland (white arrow). The arrow head shows the thinning optic nerves

**Fig. 3:**
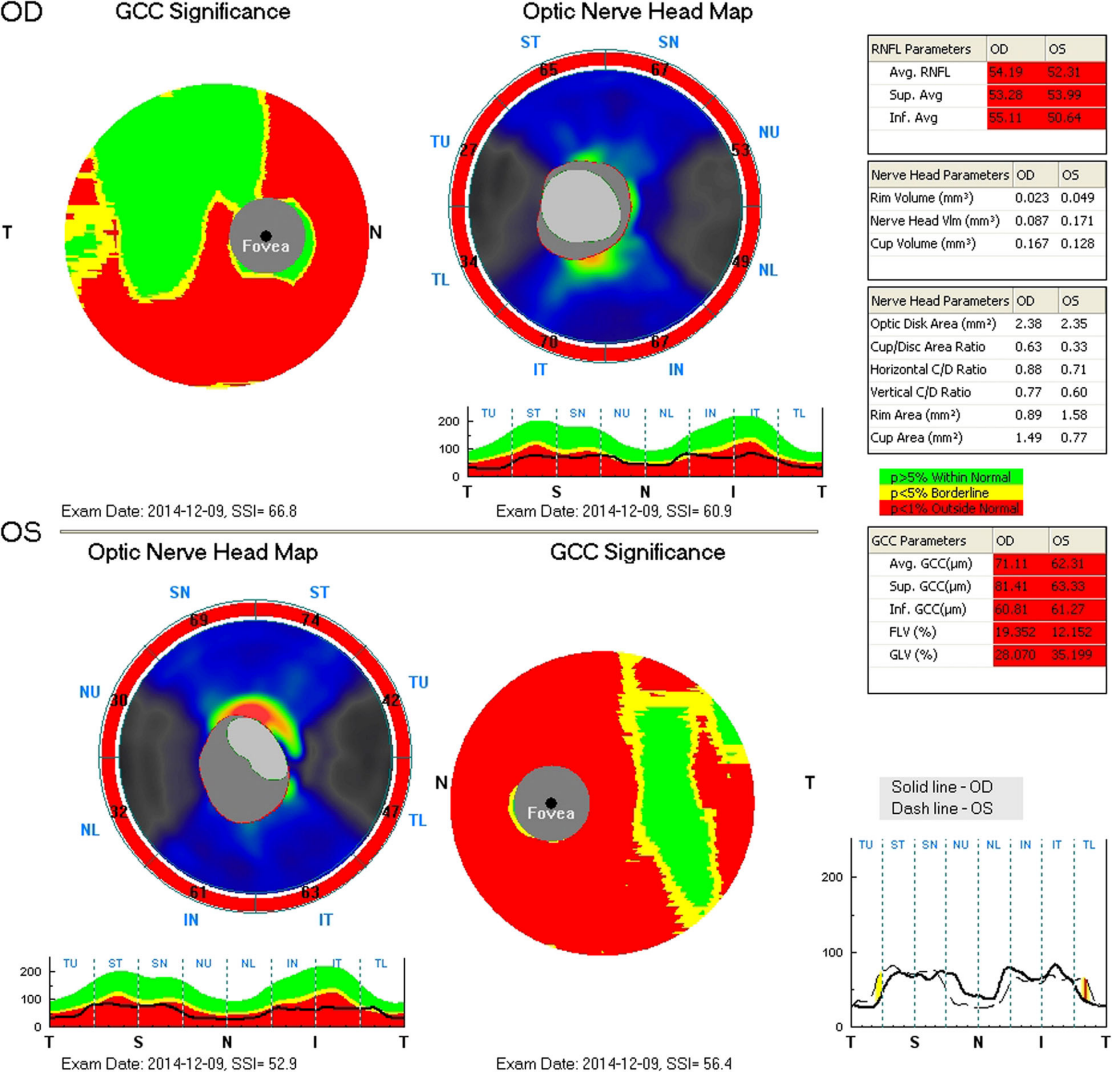
Optic coherence tomography (OCT) of the patient with *CISD2* mutation. OCT shows peripapillary retinal nerve fiber layer (RNFL) and ganglion cell lay complex (GCC) thickness significantly decreasing. The upper graphs show the thickness pattern of GCC (left) and RNFL (right) in the right eye, and the lower graphs for the left eye respectively. The red part represents the decreasing of the thickness less than 1% outside normal, and the green part indicate within the normal limit. The number labels around the optic nerve head map indicating the thickness of RNFL (μm). GCC: ganglion cell lay complex; RNFL: retinal nerve fiber layer; T: temporal; N: nasal; S: superior; I: inferior

In general, optic atrophy was observed in all four patients, demonstrated by the examination of fundus, MRI and OCT **(**Figs. 1, 2 and 3). The median age of presenting impaired vision and OA diagnosis was 12 years (range 7–39 years) and 17 years (range 7–42 years), respectively (Table 1). All patients presented severe vision loss and most of them had best corrected vision acuity (BCVA) less than 20/400 (Table 1). All of them had normal pupillary responses. All patients presented color vision loss, especially patient 1 and patient 2 had all color vision defect. Perimetry examination demonstrated various types of vision field loss, presenting as central scotomas, constriction of peripheral visual filed, segmental arcuate defect or diffuse decreased sensitivity. OCT were abnormal in all patients, showing diffused thinning of peripapillary RNFL and macular ganglion cell lay complex (GCC) (Fig. 3). ERG were normal in all patients, VEP showed latency increase and amplitude reduction in P100 waves. No one had cataract and diabetic retinopathy.

### Genetic analysis

**Fig. 4:**
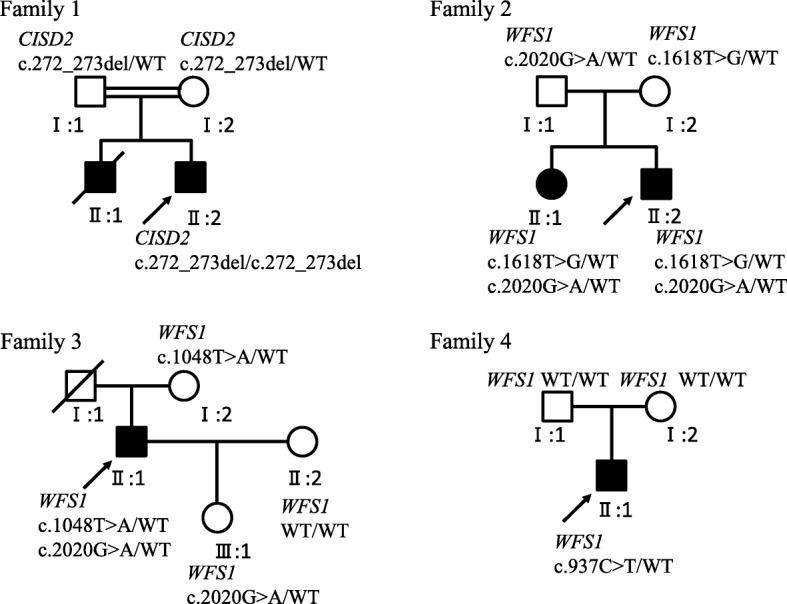
Pedigrees of four WFS families. Black squares: affected males; black circles: affected females; white squares: unaffected males; white circles: unaffected females; arrow: the proband

**Fig. 5:**
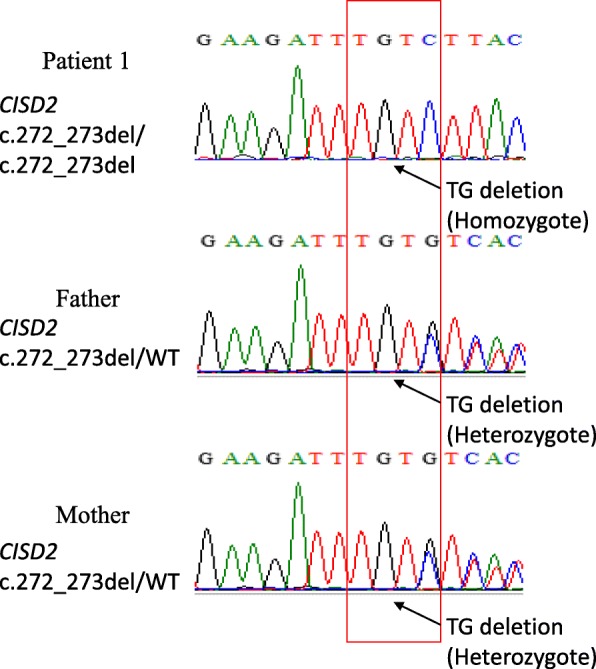
Sanger sequencing results of *CISD2* in patient 1 and his parents. Patient 1 has a novel frameshift mutation at codon 91 (p.Leu91fs) in exon 2 of *CISD2* caused by the homozygous deletion mutations (c.272_273del). The red box presents the homozygous mutation in patient 1 and the heterozygous mutation in his parents

Mutations in *WFS1* or *CISD2* gene were detected in all these patients, including one homozygous mutation on *CISD2* and four missense mutations on *WFS1* (Table 2). No other gene mutations or mitochondrial genome mutations were detected. Pedigrees with WFS in our study are shown in Fig. 4. For patient 1, we detected one novel frameshift mutation (p.Leu91fs) in exon 2 of *CISD2* caused by the deletion of two nucleotides (c.272_273del). The homozygous mutations were inherited from his parents, who were first-cousin (Fig. 5). For patient 2, compound heterozygotic mutations (c.2020G > A+ c.1618 T > G) in *WFS1* were identified and inherited from his father and mother respectively. The same mutations were also found in his sister, who had DM and OA as well (Additional file 1: Figure S1). Compound heterozygotic mutations in *WFS1* were also detected in patient 3 (c.2020G > A+ c.1048 T > A) (Additional file 2: Figure S2). Patient 4 carried a de novo heterozygotic mutation (c.937C > T) in *WFS1*, which was absent in his parents (Additional file 3: Figure S3). Four variants, including c.1618 T > G, c.1048 T > A, and c.937C > T in *WFS1* and c.272_273del in *CISD2*, were sequenced in 110 normal Chinese controls and none of mutations were detected.

**Table 2:** The mutations feature of patients with wolfram syndrome

Case no.	Gene	Nucleotide changes	Amino acid changes	Type of mutation	Zygosity	References	PolyPhen2*	SIFT**
1	*CISD2*	c.272_273del	p.Leu91fs	Frameshift	Homozygote	This study	**–**	Damaging
2	*WFS1*	c.1618 T > G	p.Trp540Gly	Missense	Compound heterozygote	This study	Possibly Damaging	Damaging
		c.2020G > A	p.Gly674Arg	Missense		[26, 27]	Probably Damaging	Damaging
3	*WFS1*	c.1048 T > A	p.Phe350Ile	Missense	Compound heterozygote	This study	Probably Damaging	Damaging
		c.2020G > A	p.Gly674Arg	Missense		[26, 27]	Probably Damaging	Damaging
4	*WFS1*	c.937C > T	p.His313Tyr	Missense	Heterozygote	[28–30]	Probably Damaging	Damaging

*PolyPhen2 Polymorphism Phenotyping 2, ** *SIFT* Sorting Intolerant from Tolerant. *SIFT* were used for the prediction of pathogenicity of all detected mutations and PolyPhen2 were used for the prediction of pathogenicity of all missense mutations

**Fig. 6:**
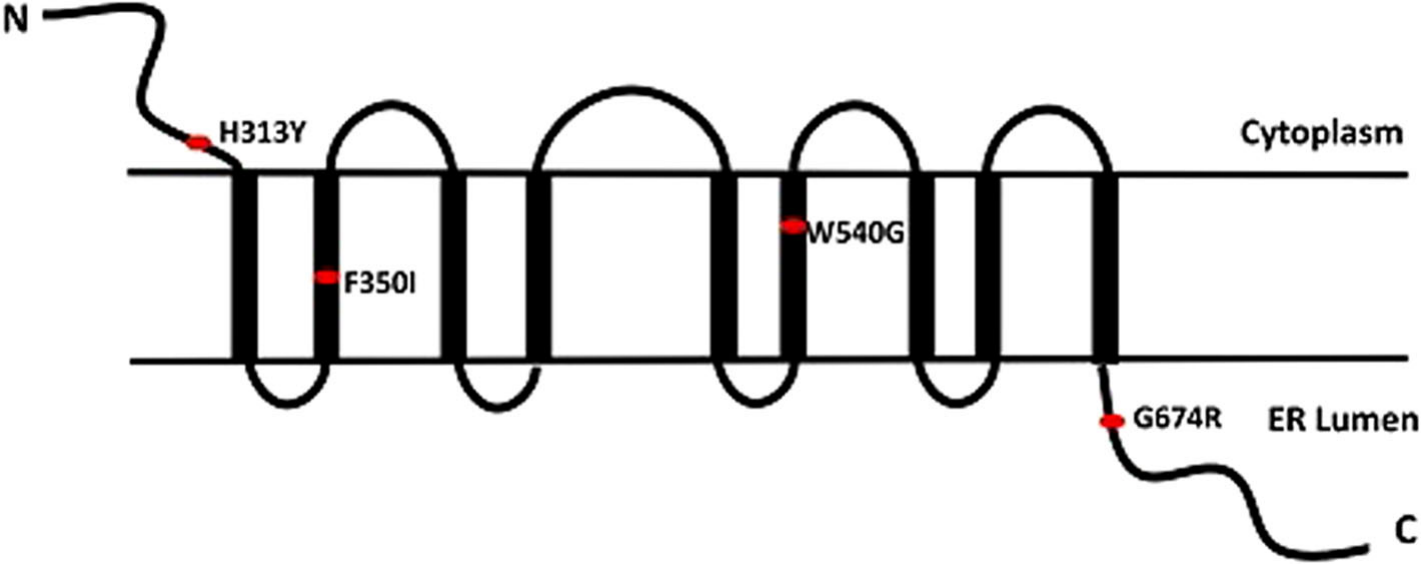
The position of the mutations in *WFS1.* Predicted structure of the wolframin with nine putative transmembrane domains, and position of the mutations in *WFS1* indicated by red cycles

Three of them were reported for the first time, including a frameshift mutation c.272_273del in *CISD2* and two missense mutations c.1618 T > G, c.1048 T > A in *WFS1*. These mutations all locate in evolutionary conserved positions of CISD2 and wolframin by multiple sequence alignment across species (Additional file 4: Figure S4). Two novel missense mutations of *WFS1* both located in exon 8 which encodes wolframin, a protein with nine predicted transmembrane domains and extracellular loops (Fig. 6). The novel variations of p.Trp540Gly (c.1618 T > G) and p.Phe350Ile (c.1048 T > A) in *WFS1* and p.Leu91fs (c.272_273del) in *CISD2* are all predicted to be highly deleterious by SIFT or PolyPhen2 (Table 2).

## Discussion

In this study, we evaluated four Chinese WFS patients and descried their ophthalmologic characteristics, as well as reported three novel *WFS1* and *CISD2* mutations. Most patients presented at least three clinical manifestations and developed at least one in their first decade, which was consistent with the systematic review of WFS [5]. A wide range of ophthalmological findings were detected including severe vision acuity lost, declined color vision, constriction of visual fields and abnormal VEP, which were consistent with previous studies [31–33]. Notably, the presenting ages of impaired vision of some patients were early than OA diagnosis age, which suggested the insidiousness of vision loss in WFS. This indicates that ophthalmologist should be aware of the possibility of WFS in young patients with severe bilateral optic atrophy. Detailed medical history inquiry and appropriate genetic testing are highly recommended for these patients.

There are two genes, *WFS1* and *CISD2,* were proven to cause WFS*. CISD2* is a rare causative gene and autosomal-recessive mutations in *CISD2* is the pathogeny of WFS2. So far, very limited mutations have been reported in this gene (Table 3) [17–20]. In our study, patient 1 was homozygous for the frame-shift mutation c.272_273del in *CISD2*, due to the parental consanguinity. This mutation was not detected in our Chinese control population. Patient 1 presented the most severe phenotype with rapid progression of disease and multisystem manifestations. The mutant *CISD2* protein exerts a deleterious influence on ER-mitochondrial structure and function and ultimately participate in multisystem neurodegeneration [20]. WFS2 firstly was regarded as a subtype which has various unique features such as peptic ulcer and bleeding tendency [17–19]. In contrast, our patient presented classical features of WFS1, including early-onset DM, progressive OA, DI and neurodegenerative features. Haematological abnormalities and peptic ulcer has not been detected so far. Our study may support the point of view that WFS1 and WFS2, caused by different genes, has a continuous clinical spectrum [20]. Since this patient was still young, with the progression of WFS2, he may develop other signs of WFS2 in the future, so long term follow-up is needed.

**Table 3:** *CISD2* mutations reported in patients with Wolfram Syndrome type 2

Gene	Population	Nucleotide changes	Amino acid changes	Exon	Consequences	Zygosity	References
*CISD2*	Jordanian	c.109G > C	p.Glu37Gln	Exon 2	Missense mutation, affects mRNA splicing	Homozygote	[19]
*CISD2*	Caucasian	intragenic deletion	–	Exon 2	Exon 2 deletion	Homozygote	[18]
*CISD2*	Italian	c.103 + 1G > A	–	Intron 1	Exon 1 be skipped	Homozygote	[17]
*CISD2*	Moroccan	c.215A > G	p.Asn72Ser	Exon 2	Missense mutation	Homozygote	[20]
*CISD2*	Chinese	c.272_273del	p.Leu91fs	Exon 2	Frameshift mutation	Homozygote	This study

Mutations in *WFS1* gene are responsible for most WFS patients. Since the discovery of *WFS1* in 1998, more than 300 different mutations have been identified in this gene [34] and majority of them located in the exon 8 encoding the nine transmembrane segments and the C-terminal tail of wolframin [33]. In this study, we found four missense mutations located in exon 8 of *WFS1*, two of them were first reported including c.1618 T > G (p.Trp540Gly) and c.1048 T > A (p.Phe350Ile). The Sanger Sequence results in control population showed that these mutations are less likely to be polymorphisms. These two novel missense mutations are located in transmembrane domain. Multiple sequence alignment showed that they were positioned within evolutionary conserved regions of wolframin. And they were predicted to be deleterious by different tools (Table 2). Notably, the mutation c.2020G > A was found in two unrelated patients in our study. This mutation was previously reported in 4 patients with DM and OA without DI and deafness [26, 27]. The allele frequency of A is < 0.0001 in Han Chinese by the 1000 Genomes Project [35]. Our result indicates that this mutation is probably a hotspot in Chinese WFS patients, which needs to be verified by more cases. Only one heterozygous mutation (c.937C > T, p.His313Tyr) was found in patient 4, which was previously detected in three patients with OA, very early DM diagnosis and profound hearing loss [28–30]*.* Coincidentally, patient 4 was diagnosed hearing loss much earlier than OA, which might provide an evidence that this mutation cause more hearing impairment than visual disability.

## Conclusions

Our study showed a group of Chinese patients with WFS who had various clinical features. Genetic analysis detected three novel mutations in *WFS1* and *CISD2*. This is the first report of Chinese patient with WFS2. Our study also illustrates the complexity and heterogeneity of WFS. So genetic testing is recommended for clinical optic nerve atrophy patients with highly suspected WFS, especially when diabetes mellitus is concomitant.

## Additional files

Additional file 1: **Figure S1.** Electropherograms of identified mutations in patient 2 and his families. (JPG 139 kb)

Additional file 2: **Figure S2.** Electropherograms of identified mutations in patient 3 and his families (his father has passed away). (JPG 133 kb)

Additional file 3: **Figure S3.** Electropherograms of identified mutations in patient 4 and his families. (JPG 56 kb)

Additional file 4: **Figure S4.** Multiple alignment of amino acid sequences of *WFS1* and *CISD2* across species. (JPG 273 kb)

## Data Availability

The datasets supporting the conclusions of this article are included within the article.

## Abbreviations

BCVA: Best corrected vision acuity
*CISD2*: CDGSH iron-sulfur domain-containing protein 2
CT: Computed tomography
D: Deafness
DI: Diabetes insipidus
DM: Diabetes mellitus
EEG: Electroencephalography
ER: Endoplasmic reticulum
ERG: Electroretinography
ERISP: Encodes endoplasmic reticulum intermembrane small protein
GCC: Ganglion cell lay complex
MRI: Magnetic resonance imaging
NGS: Next generation sequence
OA: Optic atrophy
OCT: Optic coherence tomography
OD: Right eye
OS: Left eye
PolyPhen2: Polymorphism Phenotyping 2
RNFL: Retinal nerve fiber layer
SIFT: Sorting Intolerant from Tolerant
VA: Visual acuity
VEP: Visual evoked potentials
WFS: Wolfram Syndrome
WFS1: Wolfram Syndrome type 1
WFS2: Wolfram Syndrome type 2
