# Supplementary figures and images for: Novel mutations and the ophthalmologic characters in Chinese patients with Wolfram Syndrome
Source: Orphanet J Rare Dis. 2019 Aug 7;14:190. doi: 10.1186/s13023-019-1161-y (PMC6686481; doi:10.1186/s13023-019-1161-y)

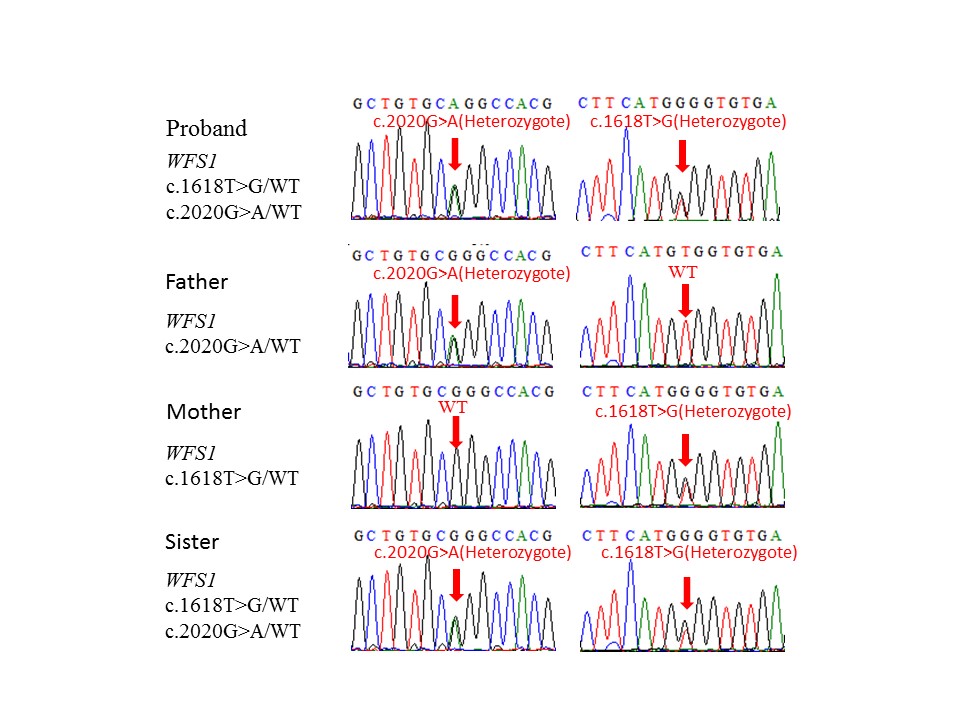

Supplement: Supplementary file 1 — Figure S1. Electropherograms of identified mutations in patient 2 and his families. (JPG 139 kb) [file 13023_2019_1161_MOESM1_ESM.jpg]

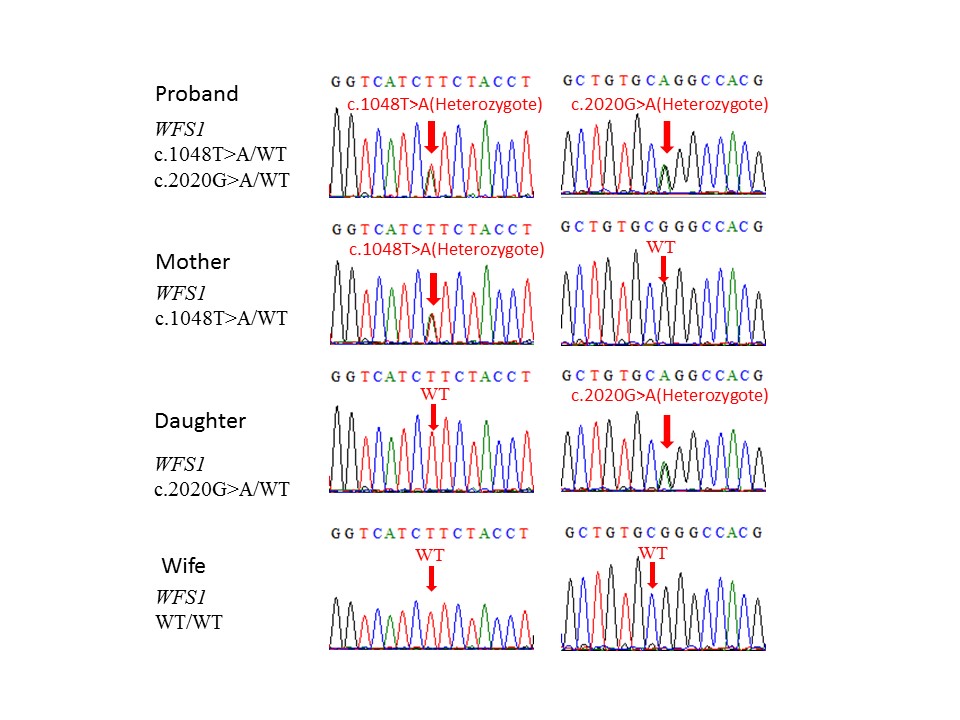

Supplement: Supplementary file 2 — Figure S2. Electropherograms of identified mutations in patient 3 and his families (his father has passed away). (JPG 133 kb) [file 13023_2019_1161_MOESM2_ESM.jpg]

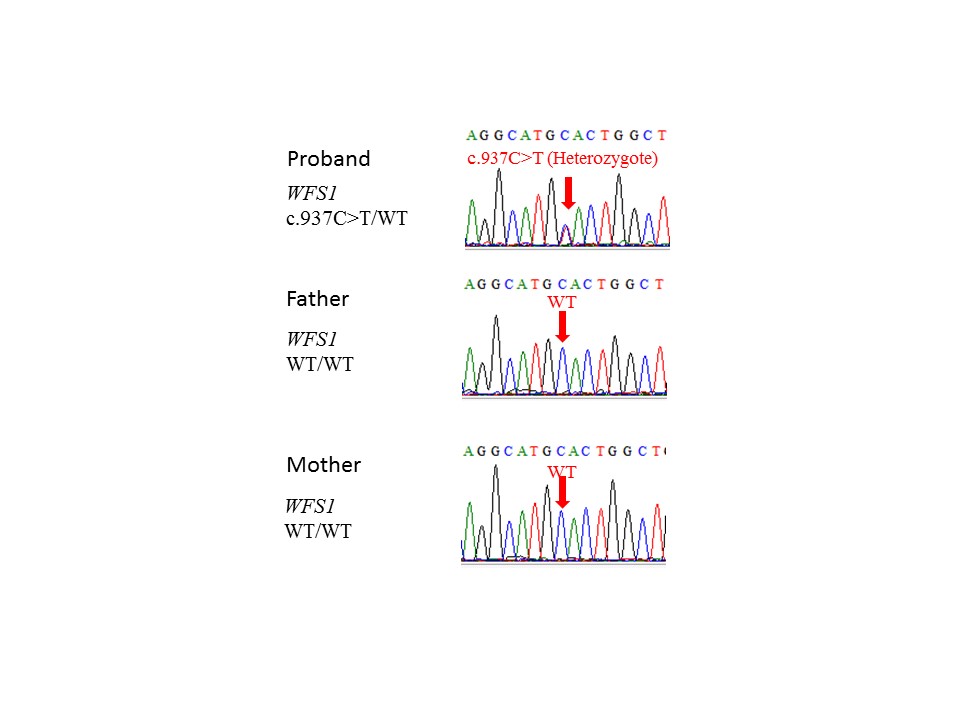

Supplement: Supplementary file 3 — Figure S3. Electropherograms of identified mutations in patient 4 and his families. (JPG 56 kb) [file 13023_2019_1161_MOESM3_ESM.jpg]

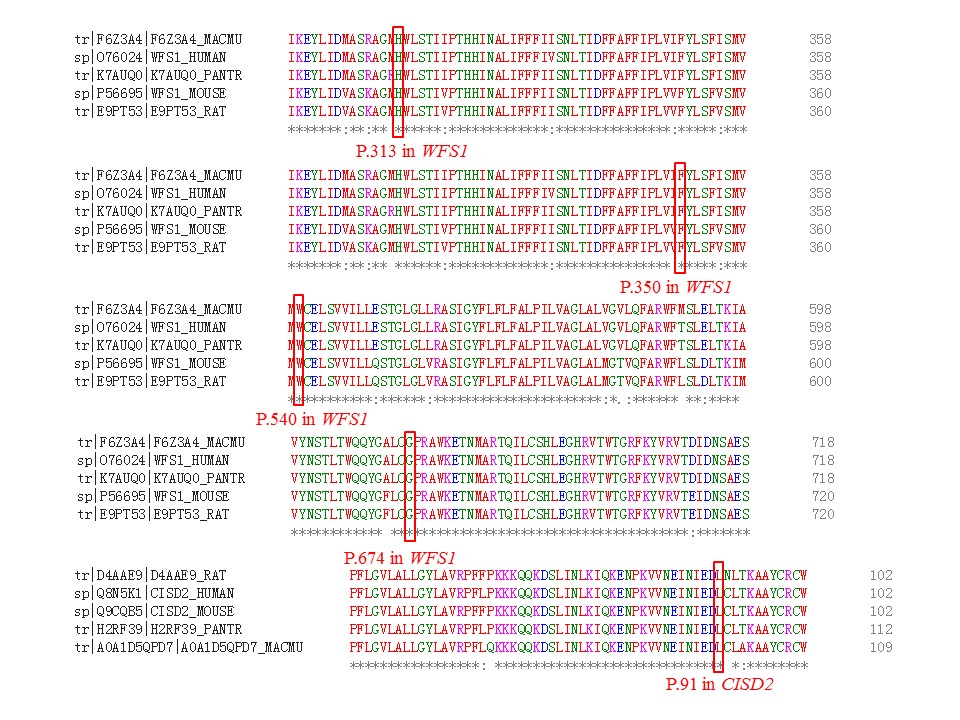

Supplement: Supplementary file 4 — Figure S4. Multiple alignment of amino acid sequences of WFS1 and CISD2 across species. (JPG 273 kb) [file 13023_2019_1161_MOESM4_ESM.jpg]
